# Clinical Application of Fiber Visualization with LIC Maps Using Multidirectional Anisotropic Glyph Samples (A-Glyph LIC)

**DOI:** 10.1007/s00062-015-0486-8

**Published:** 2015-11-27

**Authors:** M. Höller, H.-H. Ehricke, M. Synofzik, U. Klose, S. Groeschel

**Affiliations:** 1 University of Applied Sciences Stralsund, Zur Schwedenschanze 15, 18435 Stralsund, Germany; 2 Department of Neurodegenerative Diseases, Center for Neurology and Hertie-Institute for Clinical Brain Research, University Hospital Tübingen, Tübingen, Germany; 3 Department of Diagnostic and Interventional Neuroradiology, University Hospital Tübingen, MR Research Group, Tübingen, Germany; 4 Experimental Pediatric Neuroimaging, Department of Pediatric Neurology & Developmental Medicine, University Children’s Hospital, Tübingen, Germany

**Keywords:** Diffusion MRI, Line integral convolution, Visualization, HARDI, Signal processing

## Abstract

Recently, a fiber visualization method for high-angular resolution diffusion-weighted magnetic resonance imaging (MRI) data was proposed using a multiple-kernel line integral convolution (LIC) algorithm and an anisotropic spot pattern. This processing routine leads to high contrast color-coded LIC maps that are capable of visualizing local anisotropy information and regional fiber architecture. In this paper, we evaluate and validate this method by applying it to simulated datasets and to in vivo diffusion MRI data of children and adults with different disease conditions and healthy volunteers. Compared to routine clinical fiber visualization (color-coded fractional anisotropy, FA maps, and fiber tractography), it has the advantage of visualizing complex local fiber architecture in a fully automated way. The results indicate that this method is capable of reliably delineating normal fiber architecture and fibers infiltrated, displaced, or disrupted by lesions and is therefore a promising tool in the clinical context.

## Introduction

In diffusion-weighted magnetic resonance imaging (DW-MRI), the advent of high-angular resolution diffusion imaging (HARDI) techniques has triggered the development of new fiber reconstruction and visualization techniques beyond diffusion tensor imaging (DTI). Many of the limitations of the diffusion tensor (DT) model [1, 2] have been overcome by model-free representations of the local diffusion profile, for example, q-balls [3] and fiber orientation distribution functions (FOD) [4, 5]. Particularly FODs, due to their spatial sharpness, have shown good suitability to guide fiber visualization approaches, such as streamline tracking.

Fiber visualization is of great importance when cerebral lesions infiltrate, disrupt, or displace white matter tracts [6, 7]. The relationship between lesions and white matter tracts is important for understanding the functional impact of lesions, for diagnostic purposes, and for gaining a window into underlying disease pathology in poorly understood neurological diseases (e.g., complex rare neurodegenerative diseases). Moreover, it receives particular importance in neurosurgical planning. While some tumors grow and displace white matter pathways, other lesions infiltrate them. This might happen locally in the case of tumors or in a more diffuse way in demyelinating disorders like leukodystrophies.

In the latter, despite reduced anisotropy, fiber functionality might still be preserved. In other brain disorders, certain white matter pathways are selectively involved. For example, in patients with autosomal recessive spastic ataxia of Charlevoix Saguenay (ARSACS), the cortico-spinal tract (CST) was suggested to become untraceable due to the overdeveloped transverse pontine fibers [8, 9].

Line integral convolution (LIC), originally introduced by Cabral and Leedom [10], is a texture-based technique for flow field visualization. A noise input image is smoothed with a vector field using a convolution kernel which is locally adapted by vector field integration. LIC has been applied to DW-MRI data to create slice images or direct volume renderings. Hsu applied LIC to DT fields for visualization of the microstructures of tissues such as the myocardium [11]. Deoni et al. demonstrated the visualization of human brain DTI data by LIC [12]. The visualization by direct volume rendering of a three-dimensional (3D) LIC volume was proposed by Wünsche et al. [13]. HyperLIC, a multipass approach where the LIC algorithm is applied to the principal eigenvectors of the DT, was introduced by Zheng and Pang [14]. Schurade et al. combined structural and directional information by texturing a curved Klinger dissection with color-coded LIC [15]. In all of these publications on the application of LIC to DW-MRI, the DT (and only a single anisotropy direction per voxel) was used. This led to erroneous visualizations of crossing and branching pathways. Moreover, a clinical evaluation of LIC approaches to the visualization of DW-MRI has not been performed.

In Höller et al. [16], we proposed a multiple kernel LIC algorithm leading to a visualization that considers more than a single anisotropy direction. By applying it to the FOD of HARDI data, it has become possible to correctly depict fiber crossings and branchings. Using white noise as input structure for the LIC has led to a resulting image with low contrast. By the generation and usage of anisotropic cylindrical glyph samples as LIC input pattern, we were able to considerably improve contrast and significance of fiber visualizations, as compared to the previous usage of white noise [17]. The application of LIC to the FOD to visualize local fiber architecture has the potential advantage over tractography-based approaches that no user interactions (choosing seed and target regions for tracking or the manual adjustments of tracking parameters) are necessary. Also, tractography-based approaches visualize streamlines over longer distances in the brain, whereas LIC maps visualize more directly the local fiber architecture, which might be more informative in situations of local pathologies.

This paper focuses on an evaluation of the method using datasets of simulated data as well as patients with different types of brain damage and healthy controls. We then compare the outcome of our method to state-of-the-art approaches that are used in the clinical routine. These include color-coded anisotropy maps and streamline tractography [18–20].

## Subjects and Methods

Data from a simulation study and in vivo data of healthy volunteers as well as patients with different brain lesions were used. The processing and visualization was performed on a Linux (Ubuntu 14.04) workstation with an Intel Core i7 processor, 8 GB RAM, and NVIDIA GeForce GT425 graphics card.

### Simulated Data

Four synthetic diffusion datasets (12 × 10 × 14 voxels, 2 mm isotropic voxel size) containing two straight fibers (each 5 mm in diameter) crossing at different angles α (35°, 40°, 50°) were generated using the method described in Behrens et al. [21]. Two different signal-to-noise ratios (SNR = 20, 35) were modeled as complex Gaussian noise. For the generation of the synthetic datasets, 64 gradient directions were used.

### Patients

Four patients with different pathologies and two healthy control subjects were selected for this study. Their images were acquired as part of ongoing research studies approved by the ethics committee of the medical faculty of the Eberhard Karls University of Tübingen. Informed-written consent was obtained from the healthy volunteers, patients, and/or their parents.

#### P1: Fiber Displacing Tumor

Patient 1 is a 15-year-old boy with Noonan syndrome and a (medication-resistant) focal epilepsy. Since the age of 12 years, he was diagnosed with a left-sided brain tumor in the central region close to the primary motor cortex and has been evaluated for brain surgery. The boy attends normal school without any motor or cognitive deficits. Histology (after tumor resection) revealed a dysembryoplastic neuroepithelial tumor (DNET) WHO I.

#### P2: Tumor with Partly Infiltrated Fibers

Patient 2 is a 6.5-year-old boy with a brain tumor in the left central region and has been suffering from complex-focal seizures since the age of 6 years and 2 months. The boy had no further neurological symptoms and no motor or cognitive deficits. DW-MRI acquisition was performed as part of a presurgical assessment. Histology (after tumor resection) revealed a DNET WHO I.

#### P3: Selective Involvement of Fiber Tracts

Patient 3 is a 31-year-old woman with ARSACS. Her first symptoms occurred at the age of 11 years and consisted of generalized epilepsy, followed by an unsteady gait at the age of 13 years due to the development of a spastic ataxia, as well as dysphagia, dysarthria, cerebellar oculomotor dysfunction, and mild cognitive deficits. Molecular genetic testing revealed a homozygous frameshift mutation (p.Leu3102Phefs*8) in the SACS gene which is responsible for ARSACS.

#### P4: Demyelinating Lesion

Patient 4 is an 11-year-old girl with metachromatic leukodystrophy (MLD) [22]. Her first symptoms occurred at the age of 10 years and consisted of a progressively unsteady gait and deterioration of cognitive function. At the time of MRI, she underwent the Wechsler Intelligence Scale for Children and scored an intelligence quotient (IQ) of 79. She had a gross motor function measure of 100 % despite her polyneuropathy. The diagnosis was made biochemically by reduced enzyme activity of the arylsulfatase A and increased sulfatide excretion in urine. In MRI, diffuse T2-hyperintensities are thought to represent this demyelinating process [22–24].

#### C1 and C2: Healthy Volunteer

For patients P3 and P4, two healthy controls were scanned using the same sequences and acquisition parameters (Table 1). C1 was 24 years and C2 was 20 years of age. Both healthy controls were typical developmentally and were without any neurological deficits.

#### MR Techniques

The in vivo datasets were acquired on two different scanners (1.5 T Siemens Sonata and 3 T Siemens Skyra; Siemens Medical Solutions, Erlangen) at the University Hospital Tübingen. Table 1 displays an overview of the acquisition protocols.

**Table 1:** Magnetic resonance imaging (MRI) acquisition protocols

Sequence	S1	S2	S3
Scanner	Sonata	Skyra	Skyra
Magnetic field strength [T]	1.5	3.0	3.0
Number DWI directions	60	64	64
B-value [s/mm^2^]	3000	2000	3000
TR/TE [ms]	11,500/122	7300/89	15,600/101
Voxel length [mm]	2.5	2.0	1.8
Used for	P1, P2	C2, P4	C1, P3

### Data Preprocessing

For the in vivo datasets, a mutual information-based retrospective motion correction scheme was used to remove motion that occurred during the scan. For the 3 T datasets, echo planar imaging (EPI) distortion correction was used as recently described in Smith et al. [25]. For the simulated and in vivo datasets, we computed the DT and the fractional anisotropy (FA) value. One major limitation of the tensor model is that only a single principal eigenvector (representing anisotropy directions) per voxel can be identified. This is highly problematic in voxels with crossing or branching fiber populations. In the white matter of the brain, 90 % of the voxels have been shown to contain at least two different fiber populations [26]. Therefore, although widely applied in the medical field, the DT-based methods may produce false negative results in white matter regions with crossing fibers [2]. This limitation of the DT model has been recognized, and more advanced methods that overcome this limitation have been introduced [27]. Thus, we computed the FOD for each voxel with the constrained spherical deconvolution algorithm [4, 5]. We used a maximum spherical harmonic order l_max_ = 8. We determined one or two main directions for each voxel by detecting the FOD’s local maxima with a Newton–Raphson gradient ascent algorithm.

### Color-Coded FA Maps

FA maps were generated and color coded by assigning the x, y, z components of the principal eigenvector of the DT to the red, green, and blue color channels [28]. For the comparison with the LIC, the FA value was preferred to the generalized fractional anisotropy (GFA) because the GFA contrast in white matter does not show a clear advantage over the standard FA [29]. Additionally, color-coded FA maps are one of the most widely used methods for visualizing DW-MRI datasets in the clinical routine.

### Multiple Kernel LIC with Anisotropic Glyph Samples (A-Glyph LIC)

**Fig. 1:**
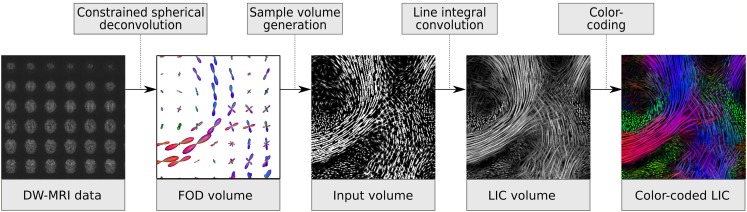
Processing steps of the A-Glyph LIC method. From DW-MRI data, the FOD volume is calculated by constrained spherical deconvolution. Then, an anisotropic glyph sample volume is generated and used as input pattern for multiple kernel line integral convolution. The LIC result is then color coded

Our method of generating anisotropic glyph samples and processing them with a multiple kernel LIC algorithm is described in detail in Höller et al. [17]. Therefore, we only present a short overview here (Fig. 1). First, a high-resolution input pattern with an isotropic voxel size of of 0.1 mm is created. Next, multi-cylindrical glyph samples derived from the FOD are placed along very short streamlines tracked over a distance in the order of the original voxel size. In this step, local anisotropy characteristics are encoded by scaling the cylindrical glyphs with the length of the FOD maxima. Subsequently, a high-resolution anisotropic glyph pattern is generated and used as input for a multiple kernel LIC algorithm. The method smooths the input pattern not only along the global FOD maxima but also along a second local FOD maximum, if present. This allows crossing and branching pathways to be delineated.

From the resulting 3D LIC volume, orthogonal slices are generated and color coded using the same color scheme as in color-coded FA maps. Additionally, color-coded LIC slices may be fused with T1 images. The processing and visualization of color-coded FA maps and color-coded LIC maps is performed by the modular software platform OpenPDT which was developed by our group.

### Streamline Tracking

Streamline tractography is certainly the most popular method for the visualization and delineation of white matter fibers. Due to the sharpness of diffusion profiles represented by FODs, deterministic tracking leads to good results even in the regions of crossing and branching pathways [30]. Probabilistic approaches may help to track through regions of low anisotropy or less distinct directions. However, tracking outcome may be even more user-dependent due to the necessity of specifying additional tracking parameters to restrict the streamline output (e.g., in- or exlusion regions for tracking). Therefore, we decided to compare our A-Glyph LIC with a deterministic tracking approach on the basis of FODs. Deterministic streamline tracking was performed on the simulated and in vivo datasets. For the simulated dataset, we used a fiber assignment by continuous tracking (FACT) algorithm implemented in our Open PDT software packages with a stepsize of 0.2 mm, a minimum GFA value of 0.1, and a minimum curvature angle of 50°/mm. Cubic seed regions of interest (ROIs) were placed manually in each of the two fibers. Within each ROI, 1000 seed points were generated. The MRtrix software package (Brain Research Institute, Melbourne, Australia) was used for the in vivo datasets. Seed regions were placed within the brain stem to track the CST and above the ventricular system to track the corpus callosum. For each seed ROI, 100,000 tracks were generated using standard parameters provided in MRtrix [30], a step length of 0.2 mm, a minimum FOD amplitude of 0.1, and a minimum radius of curvature of 2 mm. The maximum length of the track was set to 200 mm and the minimum length to 10 mm. A Newton–Raphson gradient ascent algorithm was applied to the FODs to identify the nearest peak closest to the current direction [30].

To compare tractography results with slice images (thickness: 1 mm) generated with our A-Glyph LIC algorithm, streamlines were clipped to a slab of equal size and orientation.

## Results

### Simulation Study

**Fig. 2:**
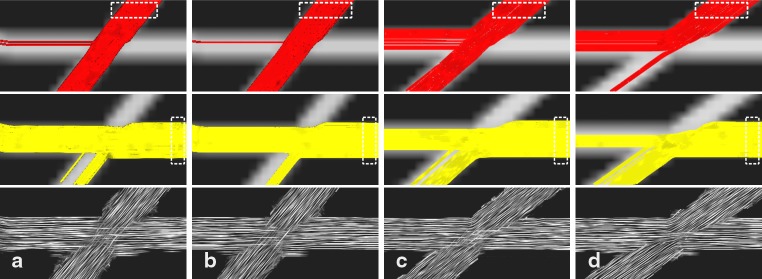
Streamline tracking results with seed ROI in upper fiber (*top row*) and seed ROI in horizontal fiber (*middle row*) and A-Glyph LIC result (*bottom row*) from simulated datasets with different crossing angles α and different signal-to-noise ratios (SNR): (**a**) α = 50° and SNR 20, (**b**) α = 50° and SNR 35, (**c**) α = 40° and SNR 35, (**d**) α = 35° and SNR 35. *Dotted rectangles* represent the seed ROI

Each of the four simulated datasets was processed with A-Glyph LIC and deterministic tracking with two different seed regions. Fibers crossings at an angle of 50° are shown in the left half of Fig. 2 with an SNR of 20 (a) and 35 (b). At a lower SNR, the number of tracks taking a wrong turn becomes higher. With A-Glyph LIC, the crossing is correctly depicted with both SNR values. The method’s robustness against noise can be explained by the smoothing characteristics of the LIC algorithm. The right half of Fig. 2 depicts results of the simulation study with lower crossing angles of 40° (c) and 35° (d) and an SNR of 35. It becomes obvious that tractography generates a great number of wrong turns, resulting from the FODs inability to correctly resolve two anisotropy directions. Some erroneous connections are visible in the A-Glyph LIC results also.

### In Vivo Study

#### Displacing Tumor, P1

**Fig. 3:**
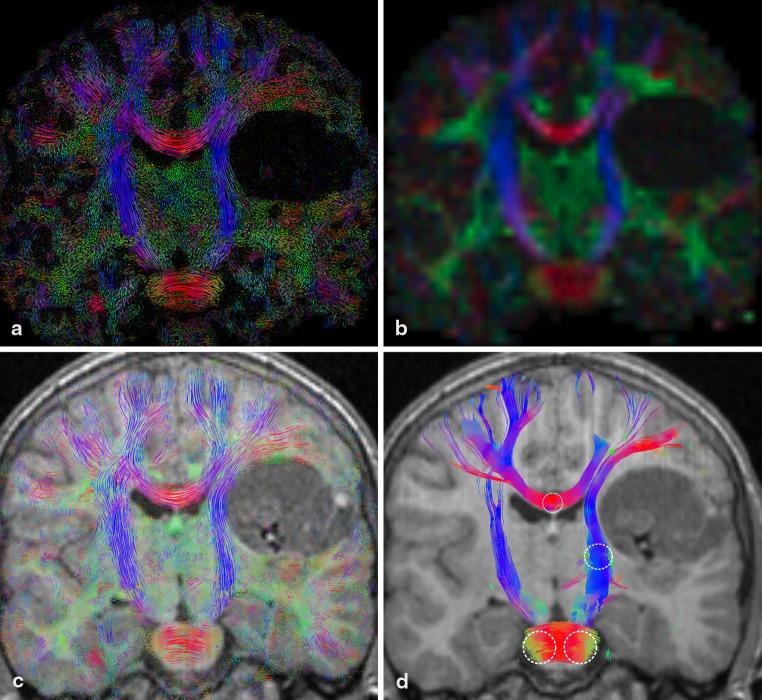
Coronal slice of tumor patient dataset (P1): (**a**) color-coded A-Glyph LIC, (**b**) color-coded FA, (**c**) color-coded A-Glyph LIC fused with T1, and (**d**) tractography result (*dotted circles* represent the seed ROI)

Figure 3 depicts four images of a coronal slice from the P1 dataset. The color-coded FA map (b) indicates a displacement of the right branch of the CST toward the ventricular system. In the crossing region of the lateral fibers of the corpus callosum, the FA map shows only a single major direction. Additionally, the FA value drops to a lower value (left side). The color-coded LIC map (a) reveals the fine structure of the underlying white matter architecture. Fibers coming out of the slice are visualized as green dots. Crossing regions can be depicted in two different ways: either between fibers both within the slice or between fibers one within the slice and one perpendicular to the slice. The LIC contains more information of the tissue structure than the FA map, especially in the crossing region of the transcallosal fibers with fibers of the fronto-occipital fasciculus and the pyramidal tracts. The main fiber architecture as depicted by the color-coded LIC (c) and the tractography result (d) is quite similar. In the tracking result, there seems to be an abrupt stop of the transcallosal fibers (d). The further course of these fibers is not shown because they extend beyond the slab.

#### Infiltrating Tumor, P2

**Fig. 4:**
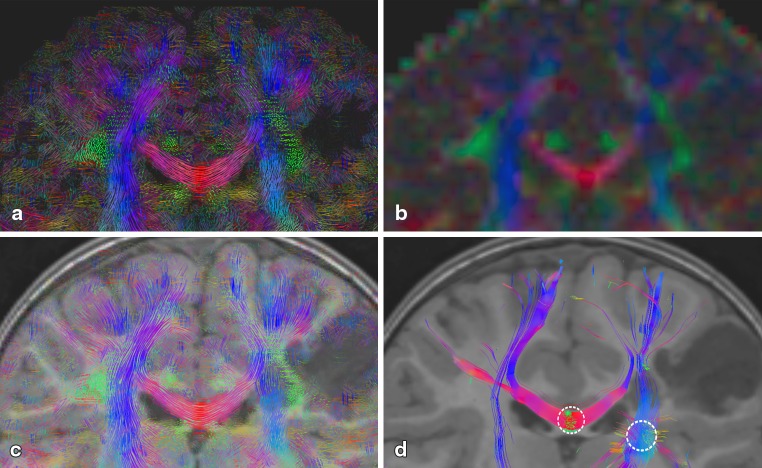
Coronal slice of tumor patient dataset (P2): (**a**) color-coded A-Glyph LIC, (**b**) color-coded FA, (**c**) color-coded A-Glyph LIC fused with T1, and (**d**) tractography result (*dotted circles* represent the seed ROI)

Four images of a coronal slice from the P2 dataset are depicted in Fig. 4. The color-coded FA map (b) shows a drop of FA in the right branch of the CST and indicates a disconnection of these white matter tracts. This is the infiltrated area of the tumor. The LIC (a and c) as well as the tractography result (d) show still existing fibers running through the affected area. Additionally, the FA value drops to a lower value (darker dropouts in the centrum semiovale). The FA map again shows only one major direction. This becomes especially evident in the area of the superior longitudinal fasciculus (SLF). The tractography result (d) does not show the SLF as no seed ROIs were placed to track this white matter tract. Both LIC results show the SLF as green dots indicating fibers coming out of the slice.

#### Disruption of Selective WM Pathways, P3

**Fig. 5:**
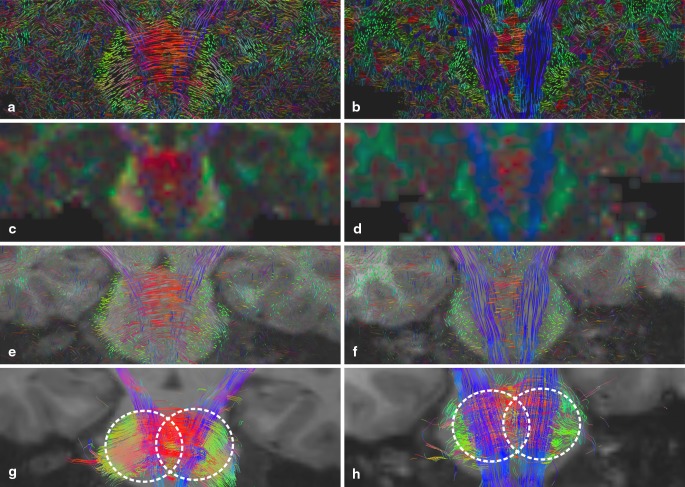
Coronal slice of ARSACS patient P3 (*left*) and healthy control C1 (*right*) from top to bottom: color-coded A-Glyph LIC map (**a, b**), color-coded FA (**c, d**), color-coded A-Glyph LIC fused with T1 (**e, f**), and tractography result (**g, h**) (*dotted circles* represent the seed ROI)

Coronal slices through the brain stem of the datasets from the healthy control C1 (b, d, e, h) and the ARSACS patient P3 (a, c, e, g) are shown in Fig. 5. In C1, the CST and the dominant fiber pathways are clearly visible. The ARSACS patient dataset shows increased prominence of pontocerebellar fibers (middle cerebellar peduncles and transpontine fibers). On the other hand, fibers of the CST seem reduced in their course but do not discontinue. This observation is both evident on the FA map (c) and streamline tracking (g) but is also clearly seen in the LIC results (a and e).

#### Demyelination, P4

**Fig. 6:**
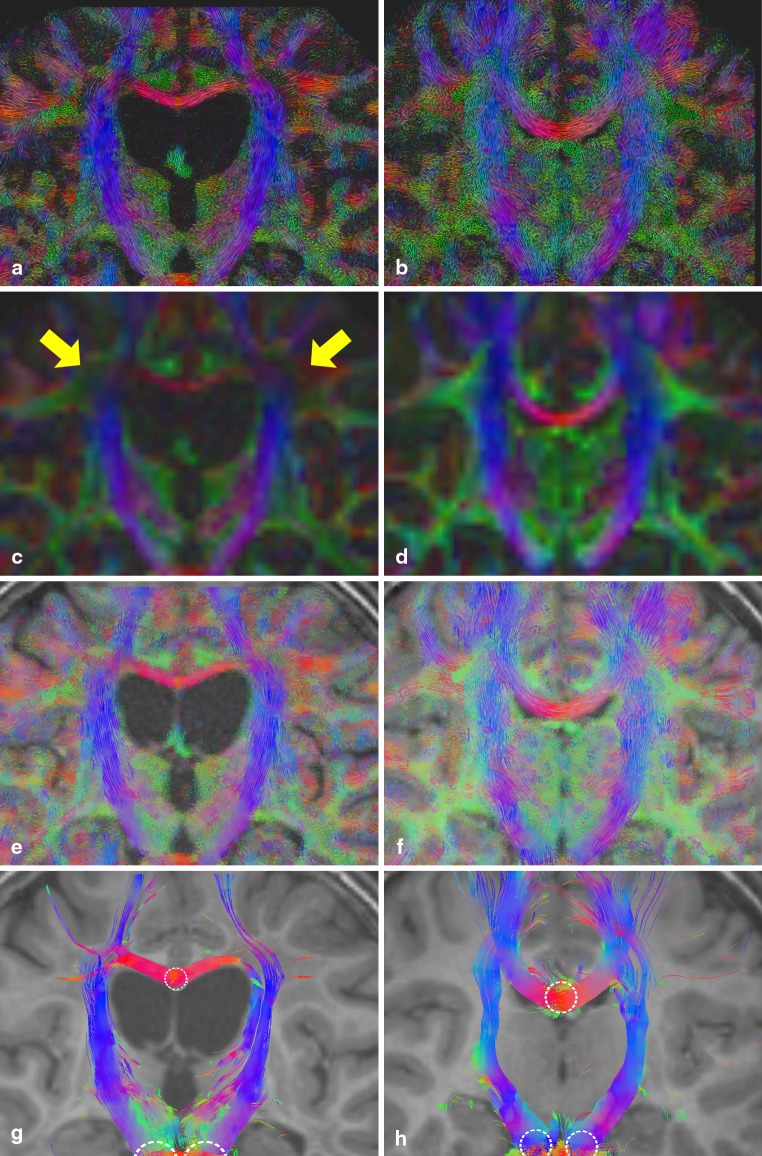
Coronal slice of MLD patient P4 (*left*) and healthy control C2 (*right*): from top to bottom: color-coded A-Glyph LIC map (**a, b**), color-coded FA (**c, d**), color-coded A-Glyph LIC fused with T1 (**e, f**), and tractography result (**g, h**) (*dotted circles* represent the seed ROI)

In Fig. 6, the results of the leukodystrophy patient dataset (left) show clear ventricular dilatation due to the diffuse white matter demyelination and consecutive atrophy of the white matter. This is also indicated by the thinner corpus callosum. The FA values of P4 in the crossing region of the corpus callosum and CST are drastically decreased (c yellow arrows). It gives the incorrect impression of a disruption of fiber pathways. This is not the case in the LIC (a and e) and the tracking result (g). As tracking is not terminated in the affected area, streamlines remain visible still running through the demyelination area. The generated LIC texture pattern clearly depicts and visualizes a loss of anisotropy in the affected region. Both the direction of FOD maxima and their amplitudes are used during the creation of the LIC input pattern. In the affected region, loss of anisotropy leads to rather low FOD maxima amplitudes, which is encoded in the LIC input pattern and consequently creates an adequate LIC texture pattern.

Furthermore, in (g) an error in the tracking result is visible. From seeds in the corpus callosum, streamlines fail to depict transcallosal fibers radiating out to the cerebral cortex, they are instead turned downward.

## Discussion

In this paper, we applied our A-Glyph LIC method to simulated datasets and in vivo datasets from patients with different brain lesions and from healthy controls. The method provided a way to visualize the known pathologies robustly and sensitively. Moreover, it already partially extended our knowledge in some of these diseases (e.g., ARSACS, see below). This might be due to the fact that compared to the widely used color-coded FA maps the A-Glyph LIC method was able to visualize local fiber architecture in more detail, especially in areas of crossing and branching fiber pathways. In contrast to previously published LIC approaches to DW-MRI, the A-Glyph LIC method works with a multiple direction convolution kernel and depicts fiber structures with higher contrast by using anisotropic glyph samples as LIC input pattern. Thus, more than a single anisotropy direction per voxel is taken into account and more complex fiber architectures can be visualized. Our results from the simulation study show that low-angle crossings down to 35− 40° can be resolved, and that the robustness against noise is better than with streamline tractography. In fact, the method is based on deterministic tracking, which is used for the creation of the anisotropic glyph samples as well as for the LIC algorithm itself. However, in contrast to streamline tractography the method applies a local tracking scheme. Tracking distances are in the order of the voxel length of the DW-MRI dataset (2.0 mm), therefore reducing the risk of error propagation during tract generation.

The method requires high-angular resolution diffusion imaging data to calculate FODs by spherical deconvolution. With newer algorithms, it is possible to generate FODs from DW-MRI data with a small number of gradients from 12 to 20 [31], and therefore data acquisition becomes easier. One of the method’s key features is its usage of a high-resolution grid, which is similar to the previously proposed track density imaging (TDI) approach [32]. In TDI, an algorithm with two main steps is applied. First, a high number (> 1.5 million) of tracks is generated using whole brain probabilistic tractography. Then, the number of tracks in each element of a grid is determined and used as input for a TDI map. The grid element can be smaller than the voxel size of the DW-MRI dataset. For comparison with our A-Glyph LIC method, we used the MRTrix software package to create a TDI map with a 0.2 mm isotropic grid. Three million tracks were generated by a probabilistic whole-brain tractography algorithm with a step length of 0.1 mm.

**Fig. 7:**
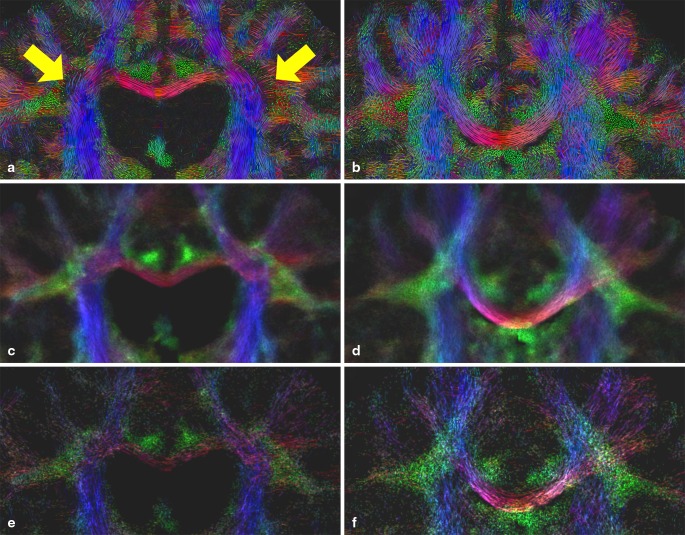
Coronal slice of color-coded A-Glyph LIC (**a, b**) and color-coded TDI map (**c, d, e, f**) of MLD patient P4 (*left*) and healthy control H2 (*right*). TDI maps generated with 3,000,000 (**c, d**) and with 200,000 (**e, f**) tracts

Figure 7 depicts a coronal slice of the in vivo datasets of P4 and C2. In contrast to the LIC result (a), the obvious loss of anisotropy in the affected region is not adequately depicted by the TDI maps (c). We assume that by the probabilistic nature of the tractography algorithm used, still a great number of streamlines is tracked through the low anisotropy region. Our efforts to minimize track density by TDI parameter tuning, particularly by increasing the FOD amplitude threshold, did not lead to better results.

In comparison to the LIC map, the structure of the underlying fiber architecture is less dominant (c, d), making the perception of fiber directions and the course of fibers more difficult. By reduction of the number of tracts in the TDI approach to 200,000, the texture of the TDI maps (e, f), looks a bit more like the LIC maps, allowing better perception of fiber geometries. However, tract densities can reliably be calculated only if a multitude of tracts have been produced, and therefore the number of tracts cannot be diminished arbitrarily.

Tumors are known to displace or infiltrate fiber pathways. The exact local relationship between the tumor and essential fiber pathways is critical (e.g., for neurosurgical planning). Therefore, fiber visualization methods must possess a high sensitivity. In our in vivo study with two tumor patients, the relationship between the tumor and the CST depicted by the LIC approach could be confirmed by the FOD-based fiber tracking method. In the case of selective changes in certain fiber pathways (ARSAC patient), the A-Glyph LIC visualization also successfully showed a difference in fiber thickness. The thinner CST due could be visualized that is despite overdeveloped pontine fibers, it was still well traceable. Thereby, this visualization method extended our knowledge on this rare, complex neurological disease. It was able to visualize in more detail the recently suspected pathology of increased thickness of both transpontine fibers as well as middle cerebellar peduncles and a reduction (not discontinuation) of CST fibers [8, 9, 33].

In the case of leukodystrophy with diffuse demyelination, the LIC method was able to visualize all fiber pathways. The color-coded FA map depicts a drastic fall-off of signal intensity in the crossing region of transcallosal and CST fibers and thus incorrectly indicates a fiber disruption. However, with A-Glyph LIC and deterministic FOD-based tractography we found that the anatomical location of fiber pathways is maintained although their density is reduced due to demyelination, as expected from pathology [22, 24].

To our knowledge, this is the first time that fiber visualization was done in MDL. From our preliminary results, it can be concluded that the method reliably visualizes different white matter pathologies and may be used in addition to or as an alternative to color-coded FA maps. Since there is no need for parameter tuning or user interaction (e.g., for the placement of seeds) and good results can be achieved with a fixed parameter set, the method is not user-dependent, does not need prior assumptions and therefore is very practical. At the same time, tractography algorithms results usually depend on user interaction and thus are less objective. An additional advantage of LIC maps is the more direct visualization of local diffusion properties in pathological brain tissue, visualizing not only local directional architecture (without the need of streamline generation over longer distances) but also local anisotropy information using the FOD maxima, which could be demonstrated in the presented cases.

The small number of patient datasets we analyzed limits the outcome of our study. Four patient datasets with different pathologies were selected and compared to healthy controls to analyze the method’s clinical usefulness. It is evident that further study with more patients and healthy controls is necessary to achieve more significant results. For this reason, we will release a freeware software package that will allow medical users to apply the A-Glyph LIC method to their DW-MRI datasets.
